# Towards a Blockchain-Based Multi-UAV Surveillance System

**DOI:** 10.3389/frobt.2021.557692

**Published:** 2021-06-15

**Authors:** Mário Gabriel Santos De Campos, Caroline P. C. Chanel, Corentin Chauffaut, Jérôme Lacan

**Affiliations:** ISAE-SUPAERO, Université de Toulouse, Toulouse, France

**Keywords:** blockchain, multi-unmanned aerial vehicles, surveillance system, embedded systems, marketplace, coordination strategy

## Abstract

This study describes a blockchain-based multi-unmanned aerial vehicle (multi-UAV) surveillance framework that enables UAV coordination and financial exchange between system users. The objective of the system is to allow a set of Points-Of-Interest (POI) to be surveyed by a set of autonomous UAVs that cooperate to minimize the time between successive visits while exhibiting unpredictable behavior to prevent external agents from learning their movements. The system can be seen as a marketplace where the UAVs are the service providers and the POIs are the service seekers. This concept is based on a blockchain embedded on the UAVs and on some nodes on the ground, which has two main functionalities. The first one is to plan the route of each UAV through an efficient and computationally cheap game-theoretic decision algorithm implemented into a smart contract. The second one is to allow financial transactions between the system and its users, where the POIs subscribe to surveillance services by buying tokens. Conversely, the system pays the UAVs in tokens for the provided services. The first benchmarking experiments show that the IOTA blockchain is a potential blockchain candidate to be integrated in the UAV embedded system and that the chosen decentralized decision-making coordination strategy is efficient enough to fill the mission requirements while being computationally light.

## 1. Introduction

Recent advances in multi-unmanned aerial vehicle (multi-UAV) or multi-robots systems in industry and research have generated many applications: surveillance (Portugal and Rocha, 2013, 2016), search and rescue (Murphy et al., 2008; Ubaldino de Souza, 2017), exploration (Benavides et al., 2019), or inspection mission (Liu and Kroll, 2012). Among them, surveillance missions are one of the most challenging since UAVs should perform persistent flight, which raises energetic and decision-making autonomy needs. Usually, UAVs fly toward Points-Of-Interest (POI) to check on it. They often embed video capability to transmit video streams in real-time or store high-quality video that can be examined *a posteriori*. Recently, these functionalities, combined with powerful machine learning features, enable UAVs to analyze the scenes themselves in order to detect anomalies (Hrabia et al., 2019).

Depending on the distance to cover, efficiency can be improved when several UAVs or robots can be used in parallel (Rizk et al., 2019). Each POI can be visited more often, leading to reducing the delay between successive visits and better surveillance service. However, the number of data exchanges and the complexity of the team coordination increase with the number of robots (Doriya et al., 2015; Benavides et al., 2019). In such cases, decentralized approaches (Cortés and Egerstedt, 2017; Ismail and Sariff, 2018) should be preferred because they prevent a single point-of-failure commonly found in centralized approaches (de Souza et al., 2016; Benavides et al., 2019). If the centralized program stops or fails, the whole system fails. The reasons causing such a failure could be multiple: the crash of the computer operating system, a loss of network connectivity, or due to external cyber-attacks such as viruses or denial of service.

Increasing the decision-making autonomy of UAVs allows to decentralize fleet management and thus increase the robustness of the global system. Many studies have considered the decentralized coordination of multi-robots (or multi-agents, or multi-UAV) for the surveillance mission (Amigoni et al., 2009; Portugal and Rocha, 2013, 2016; de Souza et al., 2016). In most of these studies, the focus is placed on the algorithms to define the cooperative behavior of the agent (e.g., UAVs, ground robots) and to coordinate movements. Aspects concerning data exchange and management are often poorly considered, and the usual hypothesis is that the data are instantaneously available to any agent at any time. This could be verified if the UAVs are permanently connected to a database (Doriya et al., 2015). If this database is centralized, this can be viewed as contrary to the objective of decentralizing the decision algorithms. If the database is replicated on several servers (possibly the robots themselves), new strong hypotheses on global synchronization and connectivity must be made (Benavides et al., 2019).

Building upon the research of de Souza et al. (2016), the objective of the present study is to propose a multi-UAV surveillance framework supported by a blockchain that could be used to provide efficient and robust data management. The proposed framework advocates how this technology can extend the services provided by such a fleet. In the game theoretical approach proposed by de Souza et al. (2016), each UAV agent chooses its next target (i.e., POI) to visit by optimizing a utility function that takes as inputs few parameters: the location of POIs, the actual known positions of the other UAV agents, and the idleness of each POI. These parameters allow the UAV agent to individually compute three utility terms, one relative to the path cost to go to a given POI, another relative to the path costs of other agents to go to a given POI, and a last one that computes an expect reward in visiting a POI, which is proportional to time passed since the last visit by any of the UAVs agents. These terms are combined in a single utility function, and the POI that minimizes such a utility function is chosen to be the next POI to visit. de Souza et al. (2016) have proven that the utility function of the complete agent, composed of the sum of the three UAVs utility functions, can be decomposed given that each individual utility function is independent from the action choices of the other UAV agents and that the game solution is a Nash equilibrium that can be obtained by optimizing the action choice of each UAV separately.

In this context, as a simple utilization of the blockchain, each agent stores in the blockchain the proof that it visits a POI and, subsequently, that its decision algorithm yields the next POI to visit. According to the context, the proof-of-visit (POV) can be a transaction signed by the agent and the POI, or if the POI cannot sign the transactions, this can be a proof-of-location (Foamspace Corp., 2018; Trouw et al., 2018; Wolberger et al., 2018). The way the proof-of-location works depends on the provider. Among the proposals cited, the approaches of Foamspace Corp. (2018) and Trouw et al. (2018) rely on physical devices that act as beacons allowing it to provide geo triangulations combined with a verified timestamp. In the case of Wolberger et al. (2018), their proposal differs from the competition by not relying on any hardware. Their method is based on sensor fusion that combines GPS, Bluetooth, accelerometers, and WiFi to determine location. The proposal has also planned to implement a reputation scoring system based on a cross-check between time and location to detect anomalies (e.g., a UAV traveling 1 km in 1 s). With such information stored in the blockchain, each agent can build its travel without additional data, relying on actual data from the UAV blockchain-supported team.

In the present study, a concept for a multi-UAV surveillance system that makes full use of the blockchain features is proposed. Interestingly, this system considers that the POIs and the UAVs are external users of the system. The features of blockchain systems, useful to deal with cryptocurrency, allowed us to design a system where every payment (transaction) is processed by smart contracts. This way, POIs can subscribe to surveillance services, and individual UAVs can participate as patrolling agents and receive a monetary compensation for that. Every time a POV is stored in the blockchain by a UAV, it leads to a transaction (in the blockchain cryptocurrency) in favor of the UAV agent. Moreover, to ensure that each UAV chooses the next POI to visit in order to optimize the global efficiency of the system (and not their personal gain), a game theoretical decision algorithm defining the next POIs is also implemented in a smart contract and thus is validated by the system. These different choices lead to a special type of surveillance service marketplace for UAVs and POIs, which ensures flexibility, security, transparency, and efficiency. The initial benchmarking experiments suggest (1) a good blockchain candidate to be integrated into UAV embedded systems; and (2) that the chosen game theoretical decision strategy can fulfill the mission requirements.

The organization of this study is as follows. In the next section, related works are reviewed, mainly about blockchains for small devices and robotics. Then, the framework proposed is presented in section 3 shows the use of a blockchain in the multi-UAV surveillance system. In section 4.1, the first blockchain implementation on small devices that would then be embedded on UAVs demonstrates that such a system is feasible. In section 4.2, a real UAVs experiment validates the decentralized mission execution control approach for the surveillance system. And, finally, the last section concludes and discusses limitations of the current work and future research directions.

## 2. Related Work on Blockchains

### 2.1. Blockchain Principle

Since the introduction of Bitcoin in 2008 (Nakamoto, 2008), the world has witnessed the large potential of such a decentralized system across many different fields. This blockchain-based approach aims to solve the major issues of centralized systems, specifically: scalability, privacy, and safety.

**Figure 1:**
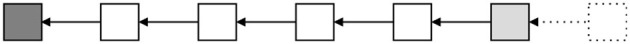
A blockchain architecture diagram. At the far left side, the dark gray represents the genesis block, i.e., the first block in the chain. The white represents four regular blocks forming the blockchain. The light gray represents the tip block, the last block of the blockchain. At the far right side, in dashed line, an example of where a potential future block can be attached is shown.

A blockchain consists of a chain of blocks connected between them by a hash. Figure 1 illustrates this concept. The dark gray represents the *genesis block*. This is the first block of the chain and holds properties like the number of tokens available in the network. The light gray represents the last block of the blockchain, the *tip block*. This is the block where a potential new block, represented in dotted style, must point to. This system implies that all participants that form the network (i.e., the network nodes) contribute to a consensus and hold a copy of the blockchain.

**Figure 2:**
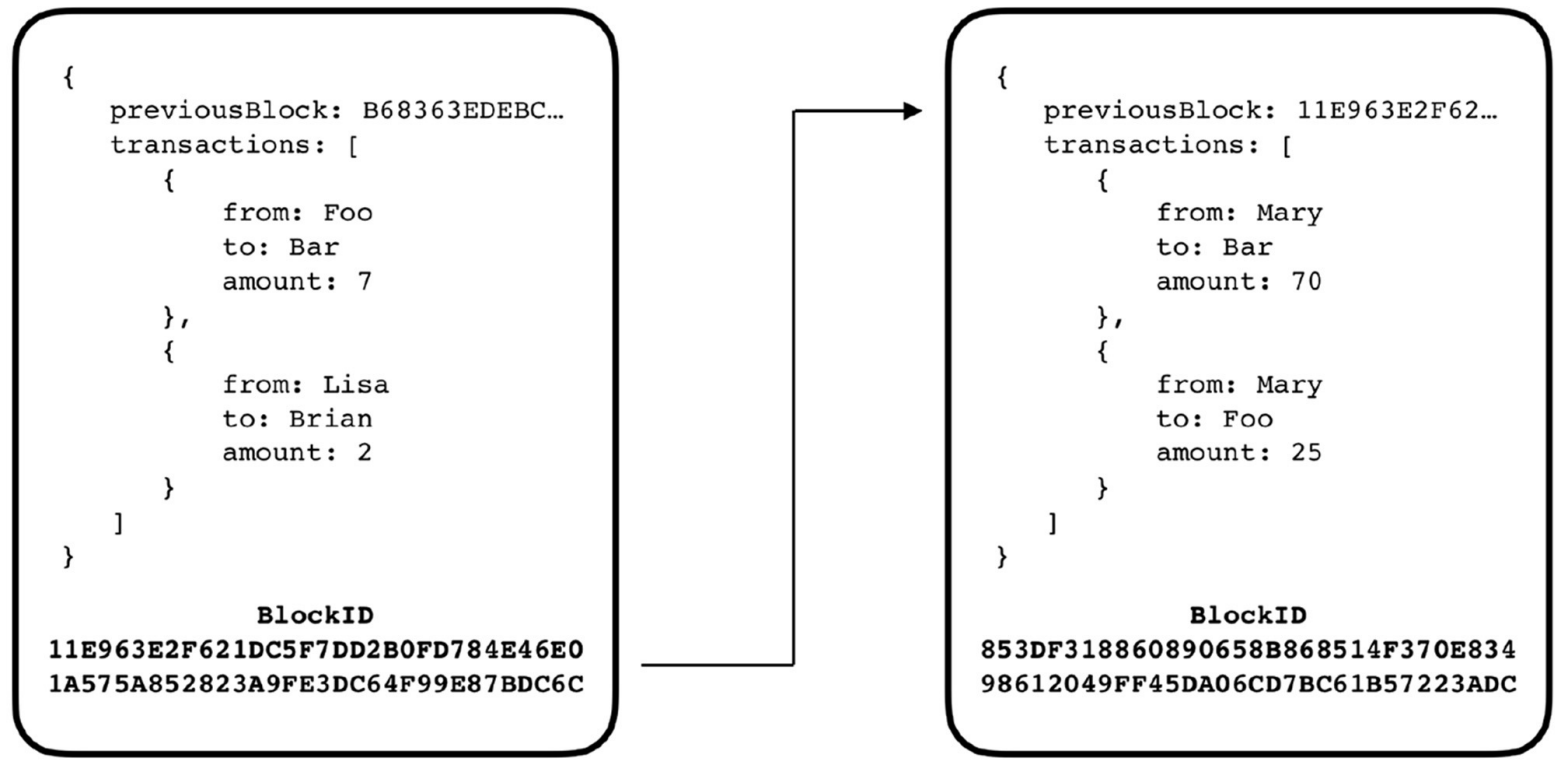
A diagram for illustrating the connection between two blocks of a blockchain. Note that the *connection* between the blocks is no more than referencing the ancestor block id. As shown, the block on the right, references the block on the left by storing its block id on the *previousBlock* key.

A block is a structured object that holds three distinct pieces of information: (i) a part that corresponds to the information that the blockchain is meant to store, like account balances and transactions in the case of cryptocurrency (represented by the key *transactions* on each block in Figure 2); (ii) the hash of the block, which the block itself points to (represented by the key *previousBlock* on each block in Figure 2); (iii) the blockID, that corresponds to the hash of the block itself, including the hash of the block which it connects to (represented in bold in each block in Figure 2). An example of a connection between two blocks of a generic cryptocurrency-based blockchain is shown in Figure 2. If a malicious entity tries to modify the content of a block, it will cause a change in the block hash and the chain will no longer be connected.

As a result of the transparency of the system and distributed validations among the network, every node needs to check the validity of new transactions added to the blockchain. The system opposition to attacks is so based on the number of nodes running the network. Each node on the network holds its own copy of the entire blockchain. Thus, in order for a certain copy to be valid, it needs to be the same across at least 50% of the network. Therefore, if a group of malicious users try to add fraudulent transactions into the network, they need to control at least half of its nodes. By Imaging a network with 10 nodes and another one with 10,000 nodes, it will be easier to manipulate the smaller network since a malicious entity would just need to add 11 nodes to have the control over more than half of it. In the case of the larger network with 10,000 nodes, it would require the addition of a significantly higher number of malicious nodes to control more than half of their nodes. Thus, the larger the network grows in terms of nodes, the more unlikely it becomes for a single malicious entity to dominate more than half of them.

To obtain the BlockID (accepted by the other members of the network), besides the computation of a simple hash, blockchains usually use a more resource-intensive algorithm. This can correspond to a simple hash criteria: for instance, imposing that every BlockID must start with four zeros (Bitcoin criteria). This approach leads to longer computations and increases the blockchain safety by increasing the time it would take to rebuild a chain. The process of computing this complex hashes is called mining.

The blockchain technology saw an expansion in its capabilities by the introduction of the concept of smart contracts, extensively used in Ethereum blockchain (Wood, 2014). A smart contract allows for distributed execution of code, and therefore, expands the blockchain capabilities further than just holding records of tokens transaction between accounts. These smart contracts can be embedded on the blockchain to increase the potential of the system to not only act as a database but also allow the storage and enforcement of these contracts.

### 2.2. Blockchains for Small Devices

Currently, almost all Internet-of-Things (IoT) devices are connected over conventional centralized networks. However, the blockchain concept presented earlier brought a new paradigm in the way these devices can communicate with each other. This approach has the advantage of being immutable to single point failure and allows immutable data sharing between entities. Moreover, the network can operate under full anonymity, which increases its safety.

Contrary to usual implementations of blockchains which use large scale clusters and powerful computers, the heterogeneous nature of IoT devices raises some challenges to blockchain implementation (IoTeX Team, 2018). The cryptographic puzzles that a typical blockchain approach solves are too complex for the CPU power of an IoT device. Furthermore, these devices are not able to store large amounts of data (in the order of dozens of gigabytes) due to their low storage capacity. For these reasons, it is impracticable to have an IoT device (e.g., UAV) performing the usual mining algorithms.

Recent studies propose mechanisms to allow the blockchain to be deployed in low power IoT devices. For instance, they can be based on the partitioning of a single blockchain network, in sub-chains, subjugated to a root chain (IoTeX Team, 2018). This hierarchic arrangement of chains that communicate with each other has the IoT devices on the bottom and powerful computers on the top (root chain).

**Figure 3:**
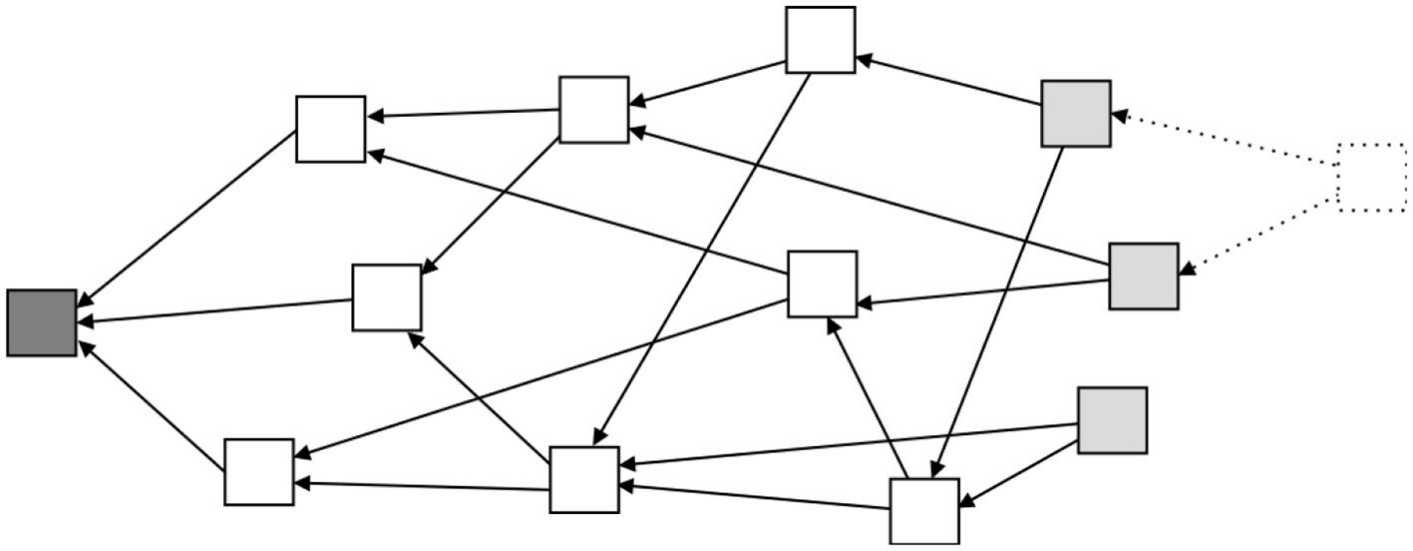
A diagram for illustrating an example of Tangle architecture. Note that, opposite to Figure 1, here one block can have more than one children. Following the same color scheme as before, dark gray represents the genesis block, white represents regular blocks, and light gray represents tip blocks. In this type of architecture, the new blocks need to attach themselves to at least two other tip blocks. This process is illustrated by the dashed line block.

Another approach to adapt blockchain concepts to small devices, called Tangles (Popov, 2018), is based on other mathematical structures. A Tangle is a directed acyclic graph, illustrated in Figure 3. This approach is defined as a natural evolution from a blockchain. By relying on a Tangle, major advantages arise such as scalability, no transaction fees, and no node discrimination. The concept is that, every new transaction (dotted block in Figure 3), when added to the Tangle, needs to connect to at least two tip blocks (light gray blocks in Figure 3). That way, this new transaction directly approves two transactions and indirectly approves all transactions that these two tip transactions point to. Since the new transaction, in order to be attached to the Tangle, needs to approve other transactions, there is no need for miners and therefore there is no node discrimination, every node publishes and also approves transactions. Consequently, since there is no need for miners, there are no associated fees with each transaction, which allows micro-payments economy. The Tangle approach is also highly scalable because the trust in a transaction is built around the number of transactions that directly and indirectly approve that transaction. For that reason, as the number of new transactions increases, the rate of growth of the trustfulness on a particular transaction also increases. Therefore, the time to approve a transaction is lower on a high load network than on a low load network. This new approach to decentralization allows to run a full node on a small IoT device. Moreover, this approach permits to run an entire network on low CPU power and low memory devices, without the need of any kind of powerful clusters to solve cryptographic puzzles.

In the following subsection, several recent studies connecting the blockchain concepts to robots and UAVs are presented. They cover most of the interests and issues relative to the use of blockchains in groups of robots.

### 2.3. Blockchains in Robotics

Ferrer (2016) proposed the first study detailing the interests of blockchains for robotic swarm systems. This study presents the integration of a blockchain in robotic swarm systems, detailing its benefits in terms of security, consensus, and transparency. It also states the drawbacks related with the difficulty of its implementation in small card computers and the increase in complexity of the overall system.

Afanasyev et al. (2019a,b) classify the blockchain-based robotics applications in their study. Among them, they identify the use of the financial side of the blockchain to implement the rewards in the case of market-based coordination strategy. They also propose to use blockchain smart contract to allocate some tasks to the robots in an unambiguous way. The system introduced in the present study can be seen as one of the applications described in these two previous studies.

The security is one the main issues in distributed systems because certain types of attacks on few nodes of a group of robots/UAVs can lead to a global failure of the network. In this vein, Kuzmin and Znak (2018) focused on blockchains embedded on UAVs and expose the main benefits from a security point of view. Strobel et al. (2018, 2020) proposes an analysis on robot-to-robot communication. The study evaluates the security and the performance of a collective decision-making scenario, connected by the Ethereum protocol. The results clearly show that, in the presence of Byzantine robots, a blockchain-based solution is much more resistant than a classical consensus algorithm. In the study presented here, security issues were not explicitly addressed because, following these two previous works, we consider that the security intrinsic to the blockchain provides this kind of service to our system.

Integrating blockchains into mobile devices could lead to network partitions which are not usual in traditional blockchains, mainly relying on wired networks. Tran et al. (2019) focused on the problems arising from swarms' partitions. In order to archive stable partitions, the SwarmDAG protocol was proposed to correctly manage the splits and merges of the network during the partitions. The study presented here considers this issue in section 3.6 by explaining how an appropriated choice of blockchain could cope with these types of issues.

A blockchain implementation in small card computers is described in Khawalid et al. (2019). There, the blockchain BigchainDB is implemented on Raspberry Pi 3B coupled with Parrot drones. The chosen blockchain has a high capacity in terms of number of transactions per second that it can handle. It was demonstrated by blending virtual and physical agents on simulation studies for validating the implementation. Despite its performance in terms of transactions rate, BigchainDB holds a copy of the entire blockchain on each node. Therefore, pursuing a more memory optimized solution while allowing a high rate of transactions, we looked for other solutions.

Falcone et al. (2019) also studied the performance aspects. The authors designed a new type of blockchain, with a specific protocol, intended for extremely low computation power devices (e.g., space applications). The authors suggest a successful implementation of that blockchain on an ARM Cortex M0 processor clocked at 48 MHz.

Kapitonov et al. (2017) linked the smart contracts of the Ethereum network and any agents which are compatible with the high-level Robot Operating System (ROS) communication framework. They also describe how this protocol can be used in a system acting as an infrastructure operator system in the field of navigation, regulatory, and economic activities using UAV. In this system, called the drone-employee project (Lonshakov, 2015), some users can rent UAV services through a blockchain located in the ground and in UAVs.

The system proposed in the present study shares several features with the one proposed by Kapitonov et al. (2017) where the blockchain is used to manage the services of a group of UAVs for some mission (e.g., filming or delivery). Since the scenario discussed in this study is more specific, surveillance scenario, we were able to go deeper in the definition of the UAV cooperation strategy and of the rewards managed by the smart contracts.

## 3. Blockchain-Based Surveillance System

### 3.1. System Hypotheses and Objectives

**Figure 4:**
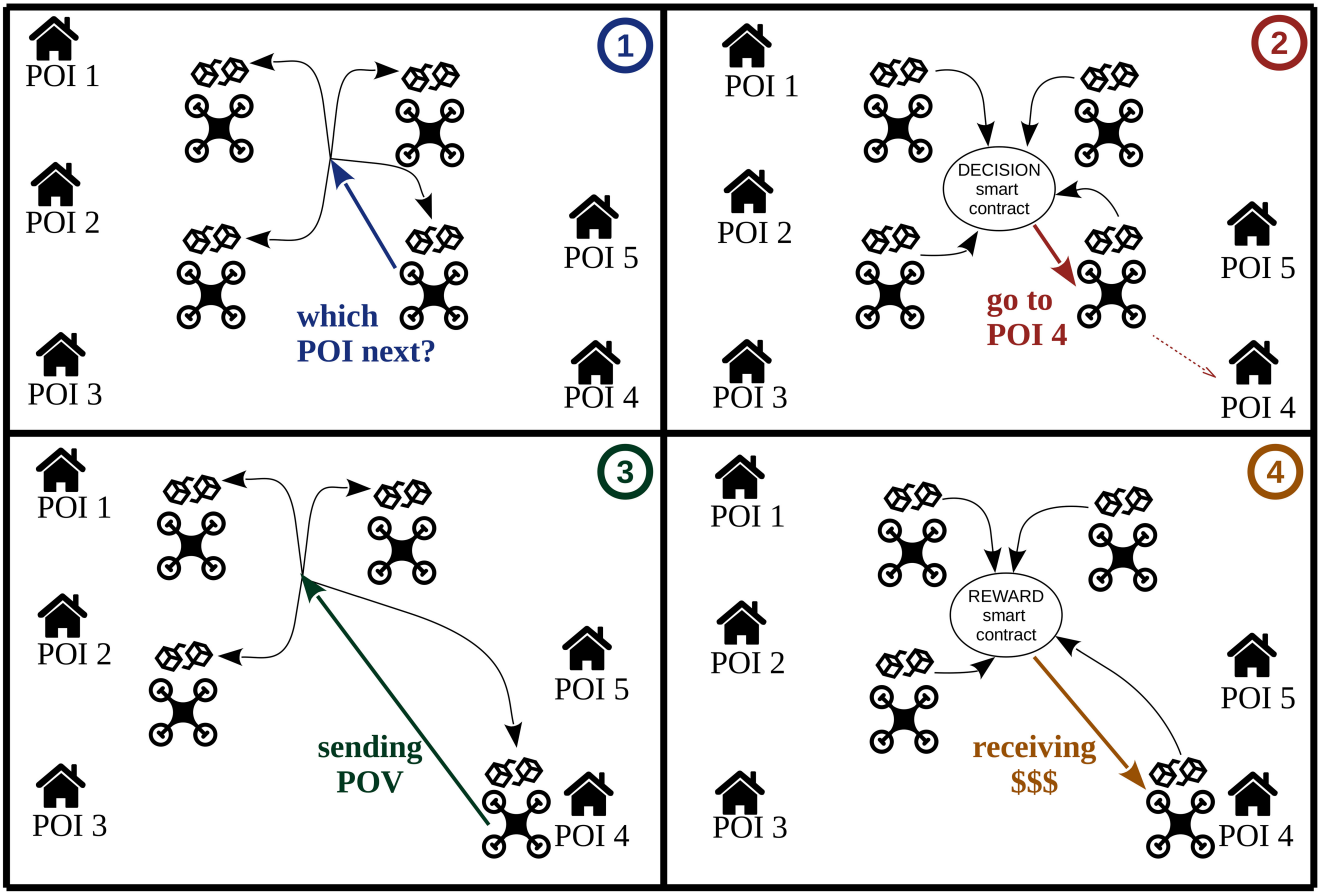
A schema of surveillance service marketplace where the UAVs are the service providers and the POIs are the service seekers. It illustrates the visit of a POI by a UAV in four steps. In step ➀, a free UAV asks to the blockchain (i.e., to all nodes in the blockchain network) which POI it must visit. The corresponding smart contract, executed by all the nodes of the blockchain, launches the decision algorithm described in section 3.3 and returns to the UAV of the chosen POI in step ➁. Then, step ➂ shows the POI visited by the UAV and produced a proof-of-visit (POV), which is stored in the blockchain. Finally, in step ➃, the smart contract managing the rewards validates the POV and pays the corresponding reward to the UAV.

In the scenario considered in this study, and illustrated in Figure 4, it is assumed that a set of independent POIs needs to be regularly surveyed by a set of autonomous independent UAVs. The system proposed here is a surveillance service marketplace where the UAVs are the service providers and the POIs are the service seekers. Note, the POIs and the UAVs are external to the system and can join and leave it at any time.

The POIs seek to be visited as frequently as possible by UAVs. For that, they buy a service to the system by paying a fee regularly which is a given amount of tokens in the system cryptocurrency. It is assumed that there exists simple ways (e.g., exchange platforms) to exchange traditional currency (dollar, euros,…) with the blockchain currency. The owners of the UAV rent their drone to the system to receive some payment in tokens. An UAV can be connected to the system as soon as it has the correct version of the blockchain software and it has an account on the system. Then, it synchronizes its local version of the blockchain and can begin to participate to the system.

The main objective of the system is to ensure a good management of the mission of the UAV and of the currency rewards in order to be attractive to both POIs and UAVs. For that, it must first be efficient in the scheduling the routes of UAV by maximizing the number of UAV visits to each POI while ensuring an unpredictable UAV behavior to prevent external agents from learning their movements and predict the future ones. Also, the system must be able to manage the potential selfish behavior of some UAV which could choose the POIs to visit in order to increase their personal gains at the expense of the global efficiency of the system. Then, it must be secure and transparent in the financial exchanges, i.e., subscription of the POIs and rewards to the UAVs.

### 3.2. High-Level Scenario Description

With respect to the presented hypotheses, we propose to build our surveillance system on a blockchain. The main advantages of this technology for robots or UAVs teams were presented in section 2.3. In this context, the most interesting features of blockchains are:

- the **network management** through a peer-to-peer structure to allow the integration or the departure of nodes;
- the **data synchronization** between the nodes through consensus protocols, which ensures the sharing of the data between all the nodes;
- the **transparency** of the rules implemented in smart contracts, which ensures the fairness between the POIs and between the UAVs;
- the **security** and the **robustness** against malicious nodes; and
- the management of **financial assets**.

The system can run on either a permissioned or permissionless blockchain. In the case of a closed system, where no direct interaction between the blockchain and the outside world is needed, the correct way to go is with a permissioned blockchain. This type of blockchain requires approval from a system manager to add new nodes to the network. In contrast, permissionless blockchains are networks where any node can freely join the system without the need for approval from a system manager. This type of approach is intended for systems that require connection with the outside world to process tasks such as payment processing.

It is assumed the users of the system are:

- the **UAVs**: They access the blockchain by reading and/or writing data. We consider that each UAV participating to the surveillance mission embeds a blockchain node. The other nodes of the blockchain are deployed on the ground. As blockchain nodes, UAVs participate in the various consensus to insert data or to run smart contracts (see Figure 4 where such actions are depicted with arrows);
- the **POI owners**: They access the blockchain to subscribe to surveillance services and to check that the service is realized. No assumptions are made on the POIs which can be simple locations or can be electronic devices. In the latter case, they can participate to the validation of the visits of the UAVs;
- the system **managers**: They access the blockchain at least to launch the system (private blockchain or application of a larger blockchain). They design and launch the various smart contracts defining the rules of the system.

### 3.3. Blockchain-Based UAV Decision-Making Coordination

In order to attract individual UAVs to participate in the system and individual POIs to subscribe it, the strategy algorithm defining each drone path must be clearly and transparently implemented on the blockchain. Thus, to ensure this property, the allocation strategy is defined as a smart contract called the decision smart contract. This implies that the algorithm is open and can be checked and validated by any user (e.g., blockchain nodes). Note that any smart contract execution is validated by a consensus between the nodes. Thus, when a UAV launches the decision smart contract to determine its next POI, the output of the smart contract can be seen as a command of the global system to the corresponding UAV (see Figure 4 steps ➀ and ➁).

#### 3.3.1. Decision-Making Strategy

The ideal algorithm to determine the UAV paths that ensures the maximum of fairness to POIs and to UAVs would be very complex (e.g., planning and scheduling approaches), which is not suitable to be implemented in smart contracts that must be run by all nodes. Consequently, the decision algorithm must be of low computational complexity in order to be implemented on a smart contract. We adopt the game theoretical decision algorithm exhaustively described in de Souza et al. (2016), because of its efficiency and its very low computational complexity.

The decision algorithm choosing the next POI is run each time a UAV has reached a POI and asks for a new one to visit. Given a UAV node, the decision algorithm computes a utility for each potential POI defined as the sum of three terms:

- the **path cost** (e.g., distance) for the UAV to move from its current position to a given POI;
- the **weighted sum of all other UAVs** ***inverted distance***, where *inverted distance* is defined as a value that is equal to the maximum distance for the nearest POI. The main objective is to make a POI that is distant from all other UAVs, more attractive to the current UAV.
- the **negative of the expected reward to reach a POI**. This expected reward value is collected (turning into zero) when a UAV passes over the position and increases by a constant factor at each time step that it is not visited. This value corresponds to the idleness of the POI. Note that this reward is a virtual value which does not correspond to any financial value.

These terms are formally defined and detailed in de Souza et al. (2016). The next POI, for a given UAV, is chosen as the one which minimize the sum of those terms. Interestingly, de Souza et al. (2016) show that the global system utility function (the sum of the UAVs individuals utility functions) is minimized when each UAV (e.g., node) selects its next POI as the one minimizing its own utility. More details concerning this property can be found in de Souza et al. (2016).

The theoretical and the simulation results of de Souza et al. (2016) demonstrated that the UAVs must not necessarily be synchronized. More precisely, the approach considered that each UAV only interacts with the others when it reaches a POI, indicating to the system that it has reached its POI. Then, it collects the data of the system it needs to compute the utility function, i.e., the last known UAVs positions (including itself), the POIs locations, and their idleness, to find the next POI. The simulation results also showed that the behavior (movements) performed by the UAVs team can hardly be predicted. It is due to the fact that if more than one POI are minimizing the utility function, a randomized choice was made. Finally, this approach scales well when the size of the robotic team increases. Those aspects demonstrate that this decision-making strategy is a good candidate to our blockchain-based surveillance multi-UAV system.

In conclusion, the adoption of this efficient allocation algorithm allows us to fulfill our first objective of maximizing the number of UAV visits to each POI, while ensuring unpredictable UAV behavior which prevents external agents from learning their movements. Those qualities, essential for a surveillance system, were demonstrated by the research of de Souza et al. (2016) in simulation. In section 4.2, we present the current implementation of this algorithm embedded in our UAVs and real-flight experiment results. Note that we assume the algorithm properties remain valid in our current implementation.

#### 3.3.2. Decision-Making Strategy Into Smart Contracts

Following the system description on the previous section, we advocate implementing it using two smart contracts, one for processing the rewards and another one for that decision algorithm (which POIs the UAVs should visit next). In this sense, the system would act in loop as follows:

1. when a UAV is idle, it asks to the blockchain which POI it should visit next (see Figure 4 step ➀).
2. then, the blockchain launches the decision smart contract to compute a next POI. When the result is validated in the blockchain, the UAV flies to the designed POI (see Figure 4 step ➁).
3. when a UAV reaches its targeted POI, it generates a POV and stores it in the blockchain (see Figure 4 step ➂).
4. next, the blockchain launches the reward smart contract which validates the visit (i.e., verifies the POV and checks that the visited POI is the one previously determined by the decision smart contract), and transfers to its account the tokens corresponding to the achieved visit (see Figure 4 step ➃).

The POV of the first point can take multiple forms. In a system without malicious users, this can simply be a statement of the UAV. However, with the considered hypotheses, a more trustworthy proof is preferable. If the POI is a connected object, a transaction signed by the POI and the UAV can be stored in the blockchain. This POV could be defined according to the context. The best solution would probably be to use the service of a location-based blockchain which provides proof-of-locations. Examples of such blockchains are FOAM (Foamspace Corp., 2018), XYO (Trouw et al., 2018), and Platin (Wolberger et al., 2018).

Concerning the second and third points, it should be noted that one of the main interests of using smart contracts is that the reward and decision procedures are executed and validated by all the blockchain nodes. This implies, for example, that a selfish UAV cannot choose individually the POI it will visit and that it must follow the output of the decision smart contract. If the UAV decides to override this decision and visit another POI, the reward smart contract will not transfer the associated tokens because the POV will not correspond to the assigned POI. These aspects allow us to fulfill another objective: to prevent a potential prejudicial selfish behavior of some UAVs.

The execution of these procedures has a relative low cost on the blockchain in terms of transactions. Indeed, for each POI visit round, one transaction is issued by the smart contract decision to determine the POI to visit, another one is sent by the UAV for the POV, and a last one is issued by the smart contract reward to transfer the tokens.

### 3.4. Financial Management

In addition to the multiple advantages of the blockchain in terms of reliability, data synchronization, and security, the blockchain can also be used to manage financial transactions between the users (that is, the main application for which it was introduced). In this case, a token is an abstraction of the cryptocurrency of the blockchain being used (e.g., Bitcoin on the Bitcoin network). We consider that the users can exchange tokens in their native currency and vice versa.

A particular type of transactions is the subscription of the POIs to the system. Practically, the POI owner transfers some tokens to a subscription smart contract which stores them on an escrow account. The transaction related to this smart contract is performed only once.

The second main financial transaction is the reward to the UAV following its visit to a POI, which is done by the reward smart contract (see Figure 4 step ➃). There are many strategies to compute the amount of tokens sent for a visit of a POI in order to fairly reward the UAVs (recall that the next POI is the result of the application of a smart contract). This analysis is beyond the scope of this study but we propose a simple rewarding strategy which consists of defining the number of rewarded tokens proportionally to the idleness. This is in accordance with the decision-making strategy presented above. Recall that the idleness is the number of time units from the last visit of the POI.

For example, let us assume that the subscription of a POI to the system is *P* tokens per time unit. If *I*(*t*) is the idleness of a POI at time *t*, we can define the reward ρ(*t*) of the UAV *i* visiting this POI as ρ_*i*_(*t*) = (1 − *T*) × *P* × *I*(*t*), where, *T* is a number in [0, 1] (possibly close to 0) corresponding to a hypothetical tax taken by the system administrators.

This type of simple strategy ensures both the financial equilibrium of the system (the escrow account has always a positive balance), a fairness between the UAVs, a stable price for the POI, and a financial feedback to the system administrators. The fairness of the service provided by the system to the POI is ensured by the strategy implemented in the decision smart contract. Note that it is interesting to observe that the financial rewards for a UAV are correlated with the virtual reward function considered by the utility function.

As for the decision-making procedures, the fact that all the different steps of the financial management correspond to transactions (tokens exchanges or calls to smart contracts) validated by the nodes of the blockchain ensures their security and transparency.

### 3.5. System Architecture

To detail the previous proposals, we present in this section, two types of architectures that are critical for the choice of the blockchain: the software architecture of the blockchain application and the embedded system architecture on the UAV.

#### 3.5.1. Software Architecture of the Blockchain Application

The blockchain-based surveillance application, we consider, corresponds to a set of specific “high-level” smart contracts that must be deployed in the blockchain to provide the services described previously:

- **subscription** smart contract: it collects the subscriptions (in tokens) of the POIs to the system in an escrow account;
- **decision** smart contract: after a UAV stores a POV, it defines the next POI by computing the one minimizing the utility functions of the UAV;
- **reward** smart contract: after a UAV stores a POV, it rewards the UAV by transferring the computed amount of tokens from the escrow account to the UAV account. If necessary, it can also transfer the tax corresponding to this visit from the escrow account to the administrator's accounts.

An intuitive illustration of the interactions between decision and reward smart contracts is given in Figure 4.

#### 3.5.2. Embedded UAV System Proposal

**Figure 5:**
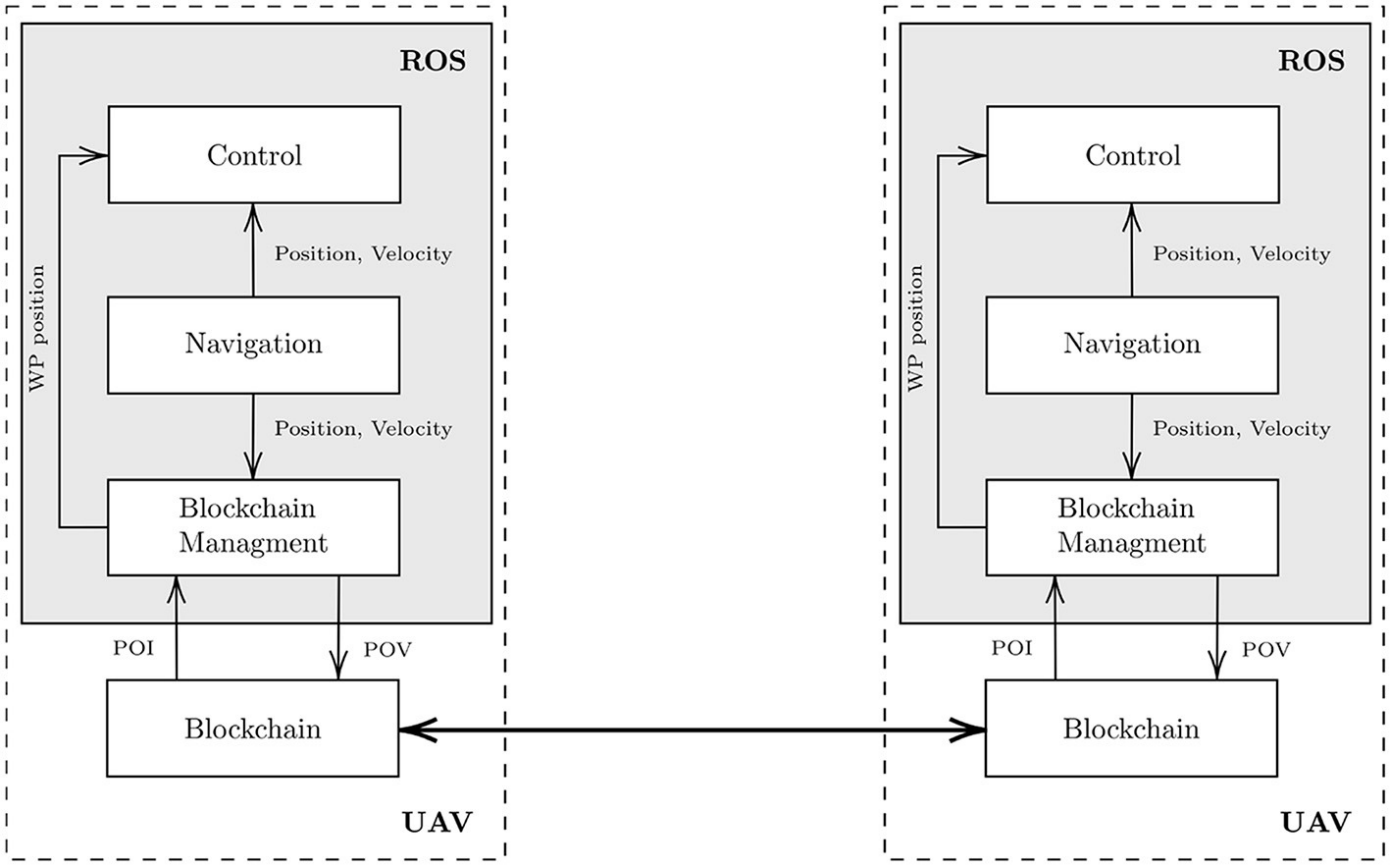
Embedded software architecture proposal. Note that this is a simplified description. For instance, we propose to integrate a Blockchain Management ROS node to ensure the dialogue with the blockchain while controlling the mission execution by sending the WayPoint (WP) position—a POI destination—to the Control node.

One of the main interests of embedding a blockchain in a multi-UAV system is to group network management, data validation, and synchronization into a blockchain that can be decorrelated from the other embedded functions. Figure 5 shows the main high-level functions suited for this application and their relationships.

The main UAV functionalities can be implemented in the ROS^1^ middleware, which simplifies the implementation of the embedded robotic software architecture. Basically, in this system, the processes are called nodes which communicate *via* a publisher/subscriber model provided by the middleware ROS. Our conceptual approach suggests implementing the system with at least the three following nodes:

- The **Navigation node** (i.e., UAV state estimation node) that provides the UAV position and velocities, which are necessary to the other nodes;
- The **Control node** (i.e., the automatic pilot node) that manages the behavior of the UAV according to the current position received from the Navigation node and to the target WayPoint (WP) position (related to the desired POI location) given by the Blockchain Management node;
- The **Blockchain management node** that makes the link between the blockchain and the other nodes:
  – It reads the POI returned by the smart contract implementing the decision algorithm in the blockchain. Then, it transmits the corresponding WP to the Control node.
  – By listening the position updates provided by the Navigation node, it detects when the UAV has reached a POI. Then, according to section 3.3, it generates a POV either cryptographically co-signed with the POI or by using a location certificate. Then, it adds this POV as a transaction in the blockchain.

The last block of Figure 5 is the Blockchain that needs to be implemented outside ROS. It is assumed to communicate with ROS nodes through a web API (REST/RPC). This block implements all the blockchain functionalities (e.g., synchronization with the other nodes, blockchain network management, insertions of new transactions, execution of smart contracts). Note that the decision algorithm is defined as a smart contract, so it can be considered as one of the services provided by this block.

It is important to observe that it is assumed the UAVs only communicate together through the blockchain. The synchronization of the blockchain clients allow to transfer the written data. This type of architecture simplifies the development of the embedded system since the embedded blockchain can be seen as a local database available to the ROS embedded robotic software architecture.

### 3.6. Choice of the Blockchain

#### 3.6.1. Types of Blockchains

The access to the system is controlled by the managers who define the list of users. As the blockchain can be either private or public, usually referred as permissioned or permissionless, there are two possible implementations of the system.

The simplest solution would be to use a permissioned blockchain dedicated to this system, for example, Quorum (Quorum Team, 2018) or Hyperledger Fabric (Androulaki et al., 2018). This type of blockchain generally offers additional services like the ciphering of the data in the blockchain which allows to control the access of each user to each data.

The other solution is to design the system as a decentralized application (Dapp) of a permissionless blockchain. In this case, the system is only defined by a set of smart contracts which manage the users and define the rules. As the smart contract would be running on a public network, the robots would also need to process transactions not only related with the patrolling mission but also with everything running on the network. This could lead to a higher CPU usage on the robots. Note that, in such a system, special attention must be given to smart contracts which are usually run by all the nodes, which could lead to a high CPU usage on the whole blockchain. Even if the security and privacy issues are harder to manage in permissionless blockchains, one very interesting advantage is the possibility to use the services of other Dapps from the same blockchain, as for instance, proof-of-locations.

#### 3.6.2. Chosen Blockchain: IOTA

The correct execution of a smart contract in a blockchain requires that multiple nodes execute the same piece of code and in the end, a consensus must be reached by a significant part of the network. The percentage of nodes that need to be in consensus and the set of nodes that will participate in the smart contract execution differs from network to network. In some cases, the number of nodes to approve a smart contract can be defined by the user issuing the smart contract, and in other approaches, this number is fixed as part of the protocol. The proportion of nodes in consensus also varies depending on the network; however, this is never <50%.

In the solution proposed in this study, the selected network is IOTA (Popov, 2018; IOTA, 2019). This offers several advantages in terms of computation power, memory consumption, and scalability, when compared with the typical blockchain approach as seen in section 2.2.

**Figure 6:**
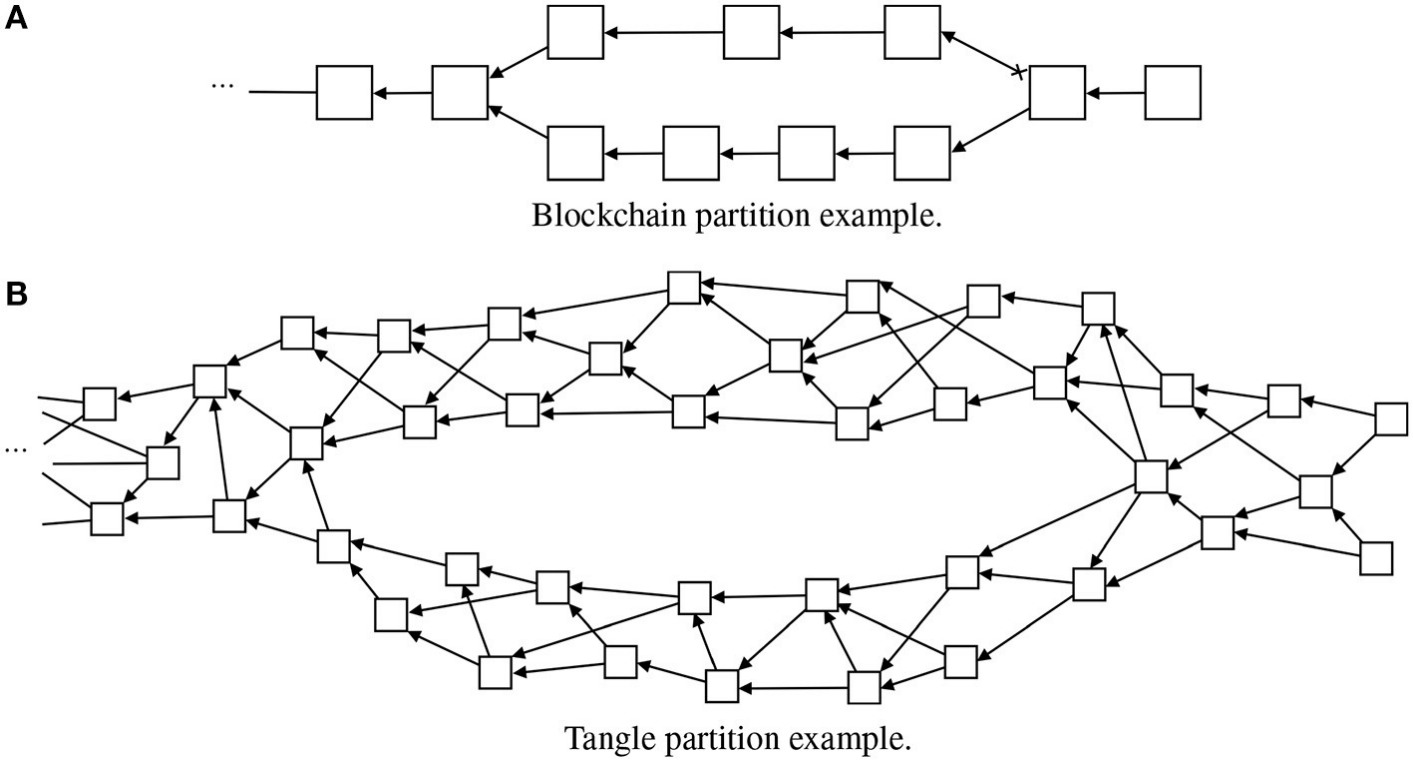
Illustration of how partitions are managed in a typical linear blockchain and in a Tangle. Note that in the blockchain case **(A)** the upper smaller chain is lost. However, in the Tangle example **(B)**, both branches are reattached without blocks loss.

In general, in a blockchain, the partitions are not possible to implement. In this case, there are two different chains in different nodes, and in the moment the network implements the merge algorithm, only the longest chain will be considered. The other chain is discarded and the blocks are deleted (see Figure 6A). However, IOTA has the ability to create partitions (iota stackexchange, 2018; Popov, 2018). Under a different approach, IOTA Tangle's can handle a partition without loss of information (iota stackexchange, 2018). This is achieved due to the nature of a tangle (see Figure 6B). If the tangle happens to be split, i.e., there are two chains that derive from the main chain and are not connected between them, there is the possibility of joining them and attaching both into a single and continuous chain again. However, the possibility of partition and reattachment afterwards only works if both sub-chains do not have transactions in conflict with each other. If there are, the transaction with the highest trust (cumulative value) will be attached to the main Tangle and the other deleted.

For a practical implementation of the multi-UAV surveillance system concept proposed, this property of reconnecting two sub-Tangles is very useful since it brings the possibility of running the application in the IOTA main-net, without overloading the network with all main-net transactions from different third parties. One of the main advantages of running the application in the main network is because that is where IOTA tokens value money and can be exchanged by any other currency like dollars and euros. However, running it simply as any other application that runs on the main-net would lead to the inconvenience of overloading the blockchain nodes embedded in the UAVs by making them approve transactions from third parties connected to the IOTA main-net.

An interesting solution would be then to create a sub-Tangle run only by the UAVs. This sub-Tangle should start by pointing to a transaction that has already been validated by a lot of transactions. This initial transaction should contain all the necessary smart contracts to launch the campaign. After that, the UAVs can perform their mission according to the algorithm presented in section 3.3 for instance. In this case, the transactions handled by the blockchain nodes embedded on the UAVs would only be related to the surveillance campaign. In the end, after finishing the mission, the sub-Tangle must be reattached to the main-net. This allows the UAVs to receive their payment in IOTA tokens that are worth *real money* and also to ensure transparency in all process. To archive this join, the sub-Tangle transactions need to be broadcasted, and, also, the tip transactions of the sub-Tangle must be promoted and then pointed by a new transaction that connects to the main-net. Since during the campaign the UAVs only broadcast and fetch data from the tangle, there is no risk of transaction conflict. After the join, the campaign missions would be available and approved by other nodes that are not UAVs. Then, the mission could be considered as validated by the global blockchain.

## 4. Experimental Results

This section presents two main experimental results. In the first part, the GoShimmer IOTA blockchain client is deployed and benchmarked on Odroid boards. In the second part, the decision algorithm is evaluated on a network of real UAVs implementing the algorithm, but without the GoShimmer IOTA blockchain. We acknowledge that we advocate the use of GoShimmer IOTA client in this study, but unfortunately, implementing IOTA blockchain for our system is currently not realizable. Indeed, the last IOTA client GoShimmer is still under development, and all its features have to be checked and integrated into the Odroid-XU4 embedded on the UAVs, in particular, the features concerning smart contracts.

### 4.1. Implementation Benchmark of the GoShimmer IOTA Client on Odroid

In order to validate the network implementation on embedded systems, a full IOTA node was deployed on a single-board computer (SBC). The chosen board was the Odroid-XU4. This board was selected due to its low cost, small size, and low power consumption. Note that this board was also chosen to be mounted in our UAVs. The used board specifications, as well as, the operating system are shown on Table 1.

**Table 1:** Odroid-XU4 board technical specifications.

**Odroid-XU4**	
CPU	Cortex^TM^-A7 Octa core
Architecture	ARMv7 Processor rev 3 (v7l)
RAM	2GB
Storage	32GB eMMC
OS	Ubuntu 16.04.6 LTS (Xenial Xerus)
Power consumption	<15 W

When compared with other similar boards in the market, as the well-known Raspberry Pi, the Odroid-XU4 outperforms them in terms of computation times and network speed, mainly due to the embedded eMMC 5.0 and Gigabit Ethernet interfaces.

The implementation used to perform the benchmarks was the IOTA GoShimmer^2^. This corresponds to a prototype of a node software that allows nodes to reach a consensus without the supervision of any third parties. The GoShimmer node software allows the creation of a fully decentralized network in which there are no discrimination amount nodes since every node can attach and also approve transactions.

On the IOTA GoShimmer node, the data is stored on a RocksDB^3^ database. This is a high-performance, non-sequential, optimized for fast storage, open-source database created by Facebook. One of the main advantages of the Tangle is that nodes do not need to hold a copy of the entire tangle. As the tangle grows, there is the option of purging a part of the tangle, i.e., the Tangle can be split and the older transactions can be deleted. Considering this operation, and in order to avoid information misses, some nodes on the network should maintain a full copy of the entire tangle. This illustrates that the Tangle is storage efficient and can run on an embedded system with just 32GB of storage capacity.

After deploying the IOTA GoShimmer on the Odroid, several benchmarks were run. The tests consist of spamming the Odroid with a constant specific number of transactions, periodically fetching the CPU load and RAM consumption. The process was repeated for different loads of transactions. In order to issue a constant number of transactions, the ZMQ spammer module available on the GoShimmer was used.

**Figure 7:**
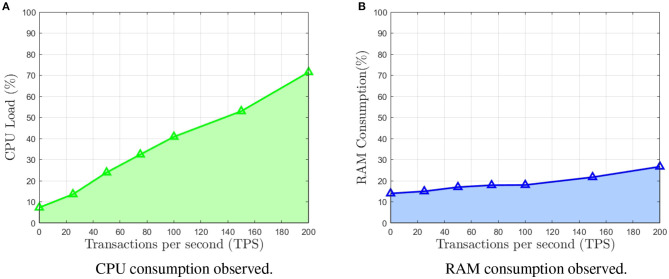
Average results for CPU load and RAM consumption of the implementation benchmark of goshimmer IOTA client on the Odroid board. Note that both metrics show a quasi-linear behavior. **(A)** CPU consumption observed. **(B)** RAM consumption observed.

The averaged results are shown in Figure 7. Both the CPU load (Figure 7A) and the RAM consumption (Figure 7B) show a quasi-linear behavior. This is expected since the addition of new transactions is of complexity O(n) (Popov, 2018). Under a load of issuing 140 new transactions per second to be approved, the CPU consumption stays around 50%.

### 4.2. Experimental Flight for Benchmarking the Implementation of the Decision-Making Strategy of the Surveillance System

In order to validate the decentralized mission execution control algorithm (described in Algorithm 1), and the related decision-making approach (mentioned in section 3.3), an implementation of the surveillance system was developed at ISAE-SUPAERO using Parrot Bebop 2 drones. This algorithm is executed in each drone, in a completely decentralized and asynchronous way, while ensuring efficient coordination to maximize POI visits and an unpredictable UAV behavior from the point-of-view of an observer, due to its properties (see de Souza et al., 2016). Note that this experimental benchmark does not use the IOTA blockchain because, even if the IOTA GoShimmer full node seems to be available, unfortunately, it is still under development and does not fulfill all the requirements for our smart contracts implementation. Therefore, the current demonstration assumes that there are no malicious UAV nodes and makes use of standard peer-to-peer communication using UDP packets. Indeed, this implementation aim is to experimentally validate the theoretical results presented in de Souza et al. (2016).

**Algorithm 1:** Pseudo-code of the mission execution control algorithm embedded in each UAV.

1: **while** True **do**
2: **if** status == *NotBusy* **then**
3: **read** messages
4: **compute** the cost for each POI
5: **select** the POI with the minimal cost strategy (see section 3.3)
6: **report** destination (e.g., next POI)
7: **assign** *Busy* to its current status
8: **start** to move to POI
9: **else**
10: **if** position == destination **then**
11: **report** destination (e.g., POV)
12: **receive** *Reward* related to the reached POI (see section 3.4)
13: **assign** *NotBusy* to its current status
14: **else**
15: **continue** to move to POI
16: **end if**
17: **end if**
18: **end while**

#### 4.2.1. The Mission Execution Control

Algorithm 1 illustrates how the mission execution control works in each UAV during the surveillance mission. Each UAV has two running statuses: *Busy* or *NotBusy*. A UAV is *Busy* (lines 9–17 in Algorithm 1) when it is navigating between POIs. When the UAV reaches its destination, it reports its status, receives the related reward (see section 3.4), and changes its statuses to *NotBusy*. When *NotBusy* (lines 2–8 in Algorithm 1) the drone computes all current costs related to all POIs being considered using the available information of its teammates and decides his next POI destination. Finally, it assigns its status to *Busy* and starts the navigation again.

The next step of this study will be to implement Tangle-based IOTA full nodes in the UAVs. We recall that the IOTA GoShimmer full node seems to be available, but unfortunately, it is still under development and does not fulfill all the requirements for our smart contracts implementation. Note that, in a future blockchain-based implementation, this execution control algorithm will be slightly modified to manage the interaction with the blockchain (see Figure 5). In other words, the mission execution control will interact with the blockchain: lines 4–6 and line 11 in Algorithm 1 will refer to a decision smart contract execution, and line 12 will refer to the reward smart contract execution.

#### 4.2.2. Flight Experiment Setup and Realization

**Figure 8:**
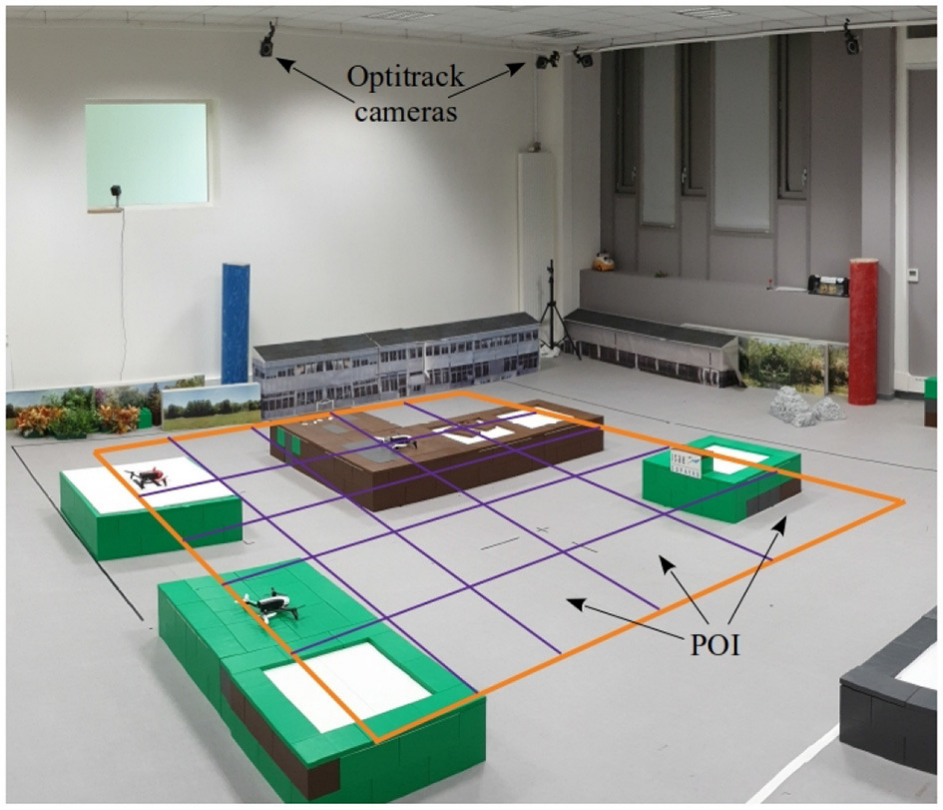
Experimental arena and facilities available at ISAE-SUPAERO. The Optitrack system allows to localize the UAVs in the arena. POIs are schematized as five-by-five grid positions.

**Figure 9:**
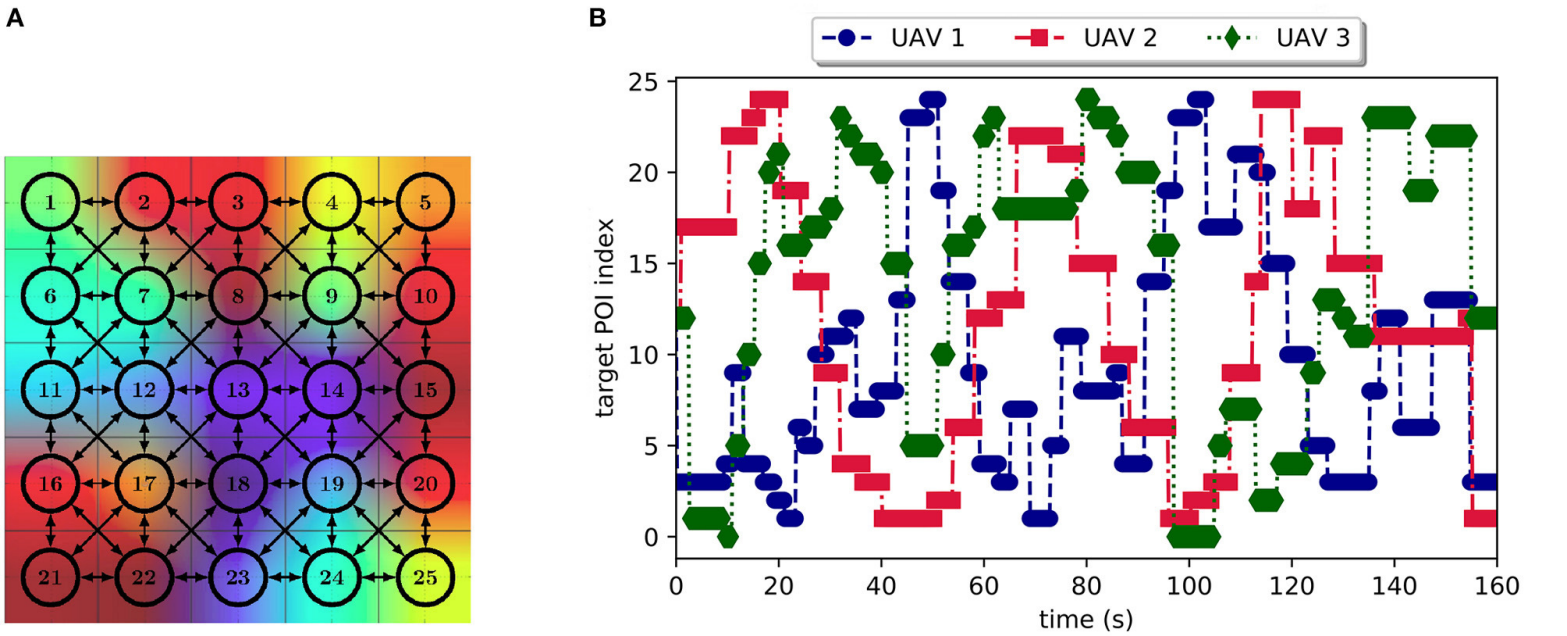
Scenario being considered during the experimental flight. The heatmap in background of **(A)** illustrates a time picture during the flight, showing the time elapsed since the last UAV visit. **(A)** Diagram of POI locations and possible paths between them used for the flight experiments. **(B)** Experiment timeline showing the different POIs visited by the UAVs. Markers show the total time elapsed to decide about POI destination and to reach the target POI.

Three Parrot Bebop 2 were used during the flight experiment represented in Figure 8. The considered arena of flight is a space of 5*m* × 5*m* to be surveyed by UAVs. This space was modeled as 25 POIs of 1*m* × 1*m*, disposed as a grid of 5 × 5 cells which is shown in Figures 8 and 9A. Only the path costs between POIs were previously computed using a Dijkstra algorithm and stored in each UAV. Note that storing path costs does not avoid the online computation of the UAV complete utility function which is minimized during decision-making process (see section 3.3). We recall that the complete utility depends on the path cost to reach a given POI; the weighted sum of inverted distance of all other UAVs, which depends on the current UAV positions; and finally, the negative of the expected reward to reach the POI, which depends on the elapsed time since the last visit of a UAV.

Since the three UAVs fly at the same altitude, an anti-collision algorithm was also implemented. This reactive anti-collision system is implemented by the Control node. The approach was to assign to each UAV another one that it should avoid. The avoidance area corresponds to a radius around the UAV in which the other UAV must avoid entering. If the flight path of a UAV coincides with the exclusion zone of the UAV it must avoid, the UAV should contour that area and then proceed to its next POI.

The UAV position was tracked during the flight experiment by a system of multiple cameras (OptiTrack system indicated in Figure 9) mounted in the lab that accurately identifies each UAV position and attitude thanks to a set of small embedded markers. This real-time tracking system allowed us to follow the path of the UAVs and to implement a POI idleness monitoring tool in the ground station. As example, the heatmap illustrated in the background of Figure 9A shows POIs being monitored during the experiment. The produced grid shows the elapsed time in each zone since the last UAV visit. The color scheme used was red for the longest time wait and blue for the shortest.

The Figure 9B presents a plot showing the experiment timeline (abscisse axis) and the different POIs (in ordinate axis) visited by the UAVs. The markers shown in this figure relate to the time elapsed between the moment a UAV decides which POI to visit (given all known UAVs current position) and the moment it reaches that POI. Please note that no cyclical behavior was observed, and all POIs were visited at least once during the flight experiment that lasted 160 s. These results validate a possible use of this decision-making strategy into smart contracts as it is computationally light and fills all mission requirements.

## 5. Discussion

We have described an innovative system allowing a set of UAVs to provide surveillance services to a set of POIs. The main technology behind the suggested system is a blockchain based on a tangle, as IOTA. The use of a game theoretical decision-making strategy for UAV coordination was validated experimentally in this study, and will be implemented on a smart contract in the future. This strategy ensures regular and difficult to predict visits of POIs by UAVs. The proposed system can be considered as a marketplace that enables POIs to buy surveillance services and UAVs to be paid for the provided service.

The Tangle-based IOTA blockchain was identified as the best candidate among the current blockchains. The first implementation tests showed that the most recent full IOTA client can be implemented on the single-board computer Odroid-XU4 and supports high transaction throughput without excessive power consumption. However, given the low number of transactions required by the proposed blockchain-based multi-UAV surveillance system concept, this consumption leaves enough processor power to run all the other algorithms, including ROS middleware implementing navigation and control modules, in the same CPU.

The implementation of the IOTA blockchain on the UAV surveillance system currently developed in the lab has yet to be completed. Indeed, the last IOTA client GoShimmer, which is promising, is still under development, and all its features have to be checked and integrated into the Odroid-XU4 embedded on the UAVs.

Several aspects of the current work can be improved or extended. The first one is the decision algorithm defining the next POIs of each UAV. The proposed game theoretical strategy taken from de Souza et al. (2016) is promising because it provides efficient results with a very small complexity. However, using a blockchain as in this system offers more possibilities than that discussed in the context of de Souza et al. (2016). Possible extensions of this algorithm could integrate constraints as, for example, maximal duration between two visits of POI due to data synchronization or due to different levels of service to the POIs. Another potential improvement of the system is the payment strategy of the UAVs, which could ensure fairness between UAVs with different levels of performance.

At the system level, it was explained that the best solution to provide trustworthy POV is the use of a blockchain-based proof-of-location. The development of such service in the suggested blockchain or the connection with other types of blockchains providing this type of service is still an open issue.

Finally, we can observe that the surveillance system has strong similarities with numerous systems of autonomous vehicles. This leads to very interesting potential system extensions. For example, the UAV system delivering packages at home is very similar to the surveillance system. Most of the system proposed in this study can be reused with minor modifications to propose a blockchain-based marketplace for package delivery by UAVs.

## Data Availability Statement

The dataset used in this article is not readily available. It is composed by the results from simulations and a flight experiment. However, any requests concerning data access should be direct addressed to the corresponding author.

## Author Contributions

This work is a joint contribution of two teams. CC and CPCC work with embedded control and automated sequential decision-making, respectively. They have chosen the decision-making strategy for the multi-UAV system and embedded the mission execution control algorithm for bechmarking the surveillance system decision strategy during the flight experiment in our lab. MS and JL develop activities on blockchains and networks. In this paper, they have designed the integration of the blockchain in the surveillance system. MS has also integrated and tested the GoShimmer client on the Odroid-XU4s. All authors contributed to the article and approved the submitted version.

## Conflict of Interest

The authors declare that the research was conducted in the absence of any commercial or financial relationships that could be construed as a potential conflict of interest.

## Footnotes

^1^https://www.ros.org/

^2^https://github.com/iotaledger/goshimmer

^3^https://rocksdb.org/
